# A Treatise on Irregularities of the Teeth and Their Correction

**Published:** 1898-04

**Authors:** 


### Bibliography.

A Treatise on Irregularities of the Teeth and their Correction. Including, with the Author's Practice, other Current Methods. Designed for Practitioners and Students. Illustrated with nearly Two Thousand Engravings (not embracing the Classification of Mechanism in third volume). By John Nutting Farrar, M.D., D.D.S., Esq., Graduate of Jefferson Medical College, Pa., and of Pennsylvania College of Dental Surgery; Member of New York Medical Society, etc. Volume II. London, New York, Leipsic: Published by International News Company.

It is rare that a reviewer, in the dental profession, is called upon to take up the examination of a book of this magnitude and one offering so many complex conditions for study. The fact that it presents over eight hundred pages and about seven hundred and fifty illustrations is sufficient indication that a superficial glance and a hasty judgment would not only be an injustice to the author, but would be equally an injury to the intelligence of the dental profession, which this work is intended to educate. An effort, therefore, has been made to examine it thoroughly, and the results might be summed up in a few words, that it stands unrivalled in profuseness of illustration and clearness of scientific statement.

The first volume deals largely with theories and principles, and the second with cases which cover a "reproduction of a mass of classified notes covering a practice of about a quarter of a century."

The author says in his "Prefatory Remarks" that "throughout the Treatise conservatism in everything has been the intention. By conservatism I do not mean 'old fogyism.' The teachings are strictly confined within scientific limits,—a range which, if understood, is intended to enable the progressive student to accomplish the best results in the easiest way with the least inconvenience to the operator and patient. . . . Effort also has been made to show in diagnosis and prognosis the distinction between that which *ought* and that which *ought not* to be attempted and that which *can* and that which *cannot* be done and that which is *possible* and that which is *best* to do."

In regard to classification the author further says, "The order of classification was mainly governed by the location of the deformity, those in the upper arch being considered before those in the lower and the anterior teeth before the posterior. For illustration the upper incisors are considered first, the cuspids next, then the bicuspids, and lastly the molars. After these the teeth of the lower jaw are considered in the same order."

The author has but little faith in teaching principles in the abstract, and asserts that it is his belief "that there can be no clear understanding of principles except through clear knowledge of details." In this he simply follows the method of the true teacher, and of which Agassiz was so strenuous an advocate, that a student must master the thing itself in all its details independent of books, principles, or technical terms. The mistake is too often made in considering all these as primarily important, relegating details to future experience. It seems, therefore, to the writer that the author founded his work on the truest principle of teaching as it is understood by the most successful educators.

In Chapter LXXI. the author details his "Philosophy and Esthetics and Theory upon Regulating Teeth." This is an interesting chapter, and should be carefully studied by the beginner in this branch of practice.

The truthfulness of the following proposition cannot be controverted and should be engraved upon every mind that first essays the moving of teeth: "If the tissue-changes can be conducted within the physiological functions, the operation of moving a tooth will be painless; but if the rate of changes be pushed beyond this condition, into the pathological, there will be more or less pain," and the author might have added, possible permanent injury to the tooth. This embodies the fundamental principle upon which the work is based, if the reviewer understands the author, and it is for this mainly that the practice so thoroughly commends itself to him. It must be a recognized fact that a tooth moved continuously and rapidly means not only destruction of surrounding tissues in their physiological relations, but a possible disturbance of function beyond the return to normality, or, in other words, a determination in the direction of the force applied that ends eventually in the very irregularity it is sought to overcome. Aside from this, it may mean devitalizing of the central organ, the pulp. Intermittent force is then the true force, periods of motion and periods of rest.

While this is true and advocated with the usual vigor of the author, he by no means confines his work to those appliances that give this intermitting force, the screw for example, but makes use of springs, rubber bands, strings, etc., where these may be safely used. It is an error, and it is thought one generally entertained, that the author repudiates all force except that which he has termed the "positive." The very opposite of this view will be exemplified in almost every chapter; in fact, the proper use of mixed appliances advocated, it is surmised, will be a revelation to some who have persistently held an opposite opinion of his work.

Criticism has been made that the author's illustrations are pen-and-ink sketches, and are not æsthetic and do not compare favorably with ordinary wood-cut engravings. This seems to the writer a very superficial view. The object of all illustrations is to render the text so clear that no mistake may be made in the author's meaning, and in the present case to enable the practitioner to follow it and manufacture the appliance exactly as represented. The illustrations drawn by him meet, beyond cavil, this demand. They are drawn with great exactness, every screw being clearly defined and the course of every thread or rubber ring so marked that the tyro can unerringly follow the course of procedure and the direction which the tooth is supposed to take. The marked difference between these illustrations, following the pen-and-ink drawings by the author, and those engraved in the usual way is plainly evident when these occur. For instance, on page 1473 an engraving is given illustrating the elevation of a cuspid by a gold mechanism. It is extremely difficult here to discover what the picture is intended to convey, and it certainly does not make the text clear. Compare this, and the one on the following page, with several in the same chapter drawn by the author and upon the same subject, and it will be clearly evident that what may be gained picturesquely by the usual wood-engraving is lost in clearness of description.

It may seem strange in an author to acknowledge ungrudgingly the merit of laborers in the same field, but it is so often neglected and that to the injury of many, that when it is found that nearly every chapter opens with a description of appliances made by others and a generous praise awarded them where it can be given, it is worthy of special notice. The author not only freely mentions this, but is quite willing to give credit even where an appliance had its origin in his own mind, but had been previously used without his having been aware of the fact. This must necessarily occur where the same train of thought has operated to produce similar mechanical fixtures. The effort to discover similarities in those used at present with those in use in the past and present century must have entailed very great labor in research. It is this that makes the work cyclopedic.

The experience of the author, covering the practice of many years, has enabled him to illustrate a variety of cases covering all possible irregularities. Fortunately for the student, these are not imaginary cases, but all with the author's sign (A) have been treated to a successful issue by the methods described. The practitioner having a given case has simply to study the teeth and illustration, and then, if so disposed, can carry on the work in the same manner. There are no superfluous words used in the text to obscure meaning. The simplicity of expression is worthy of all praise, especially in the avoidance of technical terms. This is the rock on which most beginners in teaching and writing are wrecked.

The reviewer, in passing over the pages, has found an added interest in the fact that many cases described therein come up on the tablet of memory, as though he were back in the years, endeavoring to answer the question, "How am I to regulate this case?" This query has racked the brain of many, and could these, in the past, have had this clear exemplification of how it could be accomplished, many weary hours spent in study might have been avoided. This is well illustrated in the moving of a cuspid erupting back of the central incisor, pages 1103–1117. This is, perhaps, the most difficult of all teeth to bring into alignment, at least it has given the writer more tedious discouragement than any other. The difficulties met by the author and the methods by which he finally surmounted them is, probably, as instructive as any of the cases described in the book.

Some may be inclined to doubt the possibility of making the appliances which the author describes so minutely. This will, of course, depend on mechanical dexterity. Unfortunately, all will not be equal to the work, for it certainly does require more than ordinary mechanical skill to make and place a fixture such as the writer has had the pleasure of seeing in the mouth of one of the author's patients. The perfection of adaptation and of movement is not to be obtained by indifferent mechanisms. Hence, if the volume had no other object than to illustrate a high standard of excellence, it would have performed a great service to dentistry, but this the author would probably regard as the least of its claims to consideration.

While the author in no case dogmatically assumes to dictate methods, it is evident he has his favorite modes of regulating, and is strongly inclined to the use of the screw wherever it can be applied. In Chapter CXXVIII., on the "Turning one or more Incisors by Screws," there will be found a number of twisted incisors undergoing regulation with screws placed on the labial surface of the teeth. This may seem to be open to criticism, as placing a a disfiguring appliance in full view of all observers. It must be remembered, however, that the mechanisms are drawn to an enlarged scale to show philosophy of action, while in reality they are delicately made and result in slight disfigurement.

It will probably be assumed that a multiplicity of cases would lead to a constant repetition of details. While this is, in a measure, true, the author has avoided, very happily, the monotony of illustration, and has made the book a series of cases in which each leads the student by successive steps from the simple to the complex through new adaptations of force. This is evident from the fact that interest is maintained from the opening chapter to the close, and while it is not a book to read continuously, but rather for study of details, the reader will find himself in a similar position with the reviewer, reading page upon page, each in turn revivifying old experiences and engrafting new ideas for future work.

Space will not permit further elaboration of this review. The two volumes now placed before the dental profession are models of thorough work, and, doubtless, the third volume will continue this to a completion of the author's ideal. The labor devoted to this production may find no remuneration in financial profits, and this is probably not looked for, as this labor of a life is practically a gift to the dental profession and given as an evidence of his devotion to its best interests. With this prominent idea in his mind the author has built up a work that will be for future generations a mine of scientific thought and of practical suggestions. In the reviewer's opinion it will, as the years go by, become more and more not only a work of reference, but its mechanisms will be followed just in proportion as men become more skilful in the use of tools and are able to discover the underlying principles which govern the author's work. These, based on true physiological laws, can never become obsolete, but will gain vigor as the years pass by and a higher standard prevails in dental work.

The book, it is almost needless to add, is a companion to the first volume in every respect, and both are splendid specimens of the printer's art. The author says of this, that he "has presented the work in the size and style of type that would most likely be agreeable to those members of the profession who are obliged to read evenings."

While some who entertain views antagonistic to the author may honestly criticise some things, the writer has failed, as this review demonstrates, to discover anything of vital importance with which he is not in full harmony, and hence gladly gives his full meed of praise, as worthy the subject, treatment, and the man, and it is there left for the verdict of the future.

A very full table of contents and an index of sixty-two pages, double columns, rounds out and completes the book.

The following notice of the same book was forwarded to this journal by Professor C. N. Peirce, and it is appended as part of the review:

In the first volume of this treatise, issued in 1888, embracing fourteen parts, is given a comprehensive consideration of irregularities of the teeth and the theory of their correction, including the principles of mechanism that are necessary for the execution of operations.

The second volume, now before the reviewer, embraces Parts XV., XVI., XVII. These are devoted to the application of the theories and principles of practice to which the first volume has given much space and intelligent thought. The opening of each of the parts and sections here given, as well as the initial paragraphs of many of the chapters, gives the reader an outline of the text which is to follow.

While the work, as a whole, would be by some persons thought unnecessarily large for the subject, yet the practitioner or the student who desires to find a duplicate of his case in hand, and in which he is somewhat in doubt as to the proper method of procedure, will, after carefully examining the text and illustrating cuts, be greatly assisted; moreover, he will become impressed with the aim and desire of the author to aid in the solution of the subject in many ways; so careful and clear has the author been in explaining the most important steps in operations, that the reader and student will have little or no difficulty in understanding the entire processes.

Many operations are explained in concise detail, and where the record is not so full, pictorial sketches of the principal stages supplement the deficiency in the text so well that the omission is scarcely recognized.

Throughout the treatise conservatism has certainly been the intention, for the teachings are confined within scientific limits. The operator who intelligently follows the instructions given will be able to accomplish the best results with the least amount of wasted energy, inconvenience, and discomfort to the patient as well as to himself. We have said that the volume may seem large, yet when we consider the space required for detail in order to explain the starting stages of operations, with suggestions in regard to the alterations in the mechanisms that are requisite to meet the varying changes in the positions of the teeth during the progress of the operation, the student will see little that could be omitted and yet serve his needs so well. Where age, health, and sex would seem to be an important factor in the success of an operation, the student will find timely advice.

In diagnosis and prognosis the inexperienced operator is often at a serious disadvantage. He will find most valuable assistance in this book. Between that which ought and that which ought not to be attempted—between that which can and that which cannot be accomplished—there is a range of uncertainty and speculation that to the student is not only embarrassing but almost demoralizing. By reference to the elaborate index of this volume, there can invariably be found almost duplicate cases and conditions that will be wonderfully helpful.

Every one familiar with the author's views and methods of correcting irregular teeth knows that, when it is practicable, he regards the use of power from screws as the most scientific, yet the use of elastic springs, metallic and rubber, is recognized as important adjuncts, and many such mechanisms and their method of use are illustrated.

The value of appreciating the cause of trouble is not neglected; the great importance of starting right, and in not beginning until the right line of procedure is ascertained, is strongly emphasized. To start blindly on a course, and then after futile attempts abandon the enterprise, is not only discouraging to the patient but of great disadvantage to the operator. This most unfortunate dilemma a careful study of this treatise will obviate.

The author has shown great conscientiousness in giving credit for methods suggested by other operators, and in recording the names of inventors and the dates of their inventions when possible. It would be of interest to greatly extend this notice of the work by describing some of the interesting processes, especially in elevating and in lowering teeth where desired, but that would exceed the function of this short review, the writer not hoping to do more than to interest the profession in this valuable work.

